# Combining an HA + Cu (II) Site-Targeted Copper-Based Product with a Pruning Wound Protection Program to Prevent Infection with *Lasiodiplodia* spp. in Grapevine

**DOI:** 10.3390/plants10112376

**Published:** 2021-11-04

**Authors:** Pedro Reis, Ana Gaspar, Artur Alves, Florence Fontaine, Cecília Rego

**Affiliations:** 1 LEAF—Linking Landscape, Environment, Agriculture and Food-Research Center, Associated Laboratory TERRA, Instituto Superior de Agronomia, Universidade de Lisboa, Tapada da Ajuda, 1349-017 Lisboa, Portugal; anapatriciagaspar@hotmail.com (A.G.); crego@isa.ulisboa.pt (C.R.); 2 CESAM—Centre for Environmental and Marine Studies, Department of Biology, University of Aveiro, 3810-193 Aveiro, Portugal; artur.alves@ua.pt; 3 SFR Condorcet FR CNRS 3417, Université de Reims Champagne-Ardenne, Résistance Induite et Bioprotection des Plantes EA 4707, BP 1039, CEDEX 2, 51687 Reims, France; florence.fontaine@univ-reims.fr

**Keywords:** Botryosphaeriaceae, *Trichoderma atroviride* strain I-1237, Hydroxyapatite-Copper, *Vitis vinifera*, combined strategy

## Abstract

The genus *Lasiodiplodia* has been reported from several grape growing regions and is considered as one of the fastest wood colonizers, causing Botryosphaeria dieback. The aim of this study was to (i) evaluate the efficacy of Esquive^®^, a biocontrol agent, on vineyard pruning wound protection, applied single or, in a combined protection strategy with a new site-targeted copper-based treatment (LC2017), and (ii) compare their efficacy with chemical protection provided by the commercially available product, Tessior^®^. For two seasons, protectants were applied onto pruning wounds, while LC2017 was applied throughout the season according to the manufacturer’s instructions. Pruning wounds of two different cultivars were inoculated with three isolates of *Lasiodiplodia* spp. Efficacy of the wound protectants, varied between both years of the assay and according to the cultivar studied but were able to control the pathogen to some extent. The application of LC2017 did not show clear evidence of improving the control obtained by the sole application of the other products tested. Nevertheless, LC2017 showed a fungistatic effect against *Lasiodiplodia* spp., in vitro, and has previously shown an elicitor effect against grapevine trunk diseases. Therefore, this combination of two protection strategies may constitute a promising long-term approach to mitigate the impact of Botryosphaeria dieback.

## 1. Introduction

Botryosphaeria dieback is currently among the most significant grapevine trunk diseases (GTDs) in all the grape-growing regions of the world [1]. It represents one of the major threats to sustainable and economically viable viticulture due to the reduction in yield, increased crop management costs, and shortened life span of vines and vineyards [1,2,3]. This disease is caused by fungi of the family Botryosphaeriaceae and more than 26 taxa of this family have been associated with Botryosphaeria dieback in grapevine [4,5,6,7,8]. *Lasiodiplodia* spp. and *Neofusicoccum* spp. were previously proven as being amongst the fastest wood colonizing genera, and therefore considered as some of the most virulent GTD fungi [9,10,11,12]. The genus *Lasiodiplodia* comprises 34 species [13,14], from which ten have been reported from the grapevine [13,14,15,16,17,18,19]. *Lasiodiplodia theobromae* is the most commonly isolated species in grapevine and, although it is most common in tropical and sub-tropical regions, it can be found in vineyards around the world, such as Australia [20], Algeria [21], Brazil [17], Bolivia [22], China [19], Italy [23], Mexico [9], Peru [24], Portugal [25], Spain [26], Turkey [27], and the USA [9]. Common external symptoms caused by infection with botryosphaeriaceous fungi on grapevine, include leaf spots and wilting, dry fruit rots, bud necrosis and perennial cankers, cordon dieback, and eventually the sudden death of the plant [7,28,29]. The internal wood symptoms usually consist of wedge-shaped necrotic sectors and brown streaking below the bark, sometimes beginning in the pruning wounds [30]. These fungi are air-borne and infect grapevine through any type of wound, but primarily the infection occurs through the pruning ones [1]. Pycnidia of different Botryosphaeriaceae species associated with Botryosphaeria dieback can be found within old pruning wounds, in dead or cankered wood, embedded on the bark of cordons and trunks of infected grapevines. They can also be found on pruning debris left in the vineyard, constituting a potential source of inoculum for new infections [7,31,32]. Up to now, there are no curative methods to mitigate infection by Botryosphaeriaceae species. Preventive control methods such as pruning wound protection are currently the practice proving to be more efficient [1,28], especially if carried out from the early stages of the vineyard lifespan [3,33]. The field efficacy of chemical wound protectants against botryosphaeriaceous fungi has been demonstrated in several grape-growing regions of the world, namely Australia [34], Chile [35], New Zealand [36,37], Portugal [38], South Africa [39,40], Spain [41], and the USA [42]. Another method considered to be the most effective strategy for controlling infection by GTD pathogens is the application of pastes and paints amended, or not, with fungicides. This latter can provide a physical barrier, preventing spore germination to occur in the wound, but if this barrier is altered by any external factor, the supplementation with a fungicide will act on the pathogen, inhibiting its growth [1]. Their efficacy has also been shown, specifically against Botryosphaeriaceae [34,35,41,42,43]. The currently existing active ingredients (AIs) are effective in protecting pruning wounds but have limited systemic activity. These AIs usually do not penetrate well enough into the grapevine tissues to effectively control pathogens inside the vascular system [28,44] and limit the colonization by pathogens. Therefore, new methods to efficiently deliver fungicides to specific targeted areas of the plant are considered to have great potential to improve GTD control. The application of site-targeted fungicides to protect vascular tissues against GTD pathogen colonization has been recently investigated by several authors [44,45,46]. Lignin nanocarriers loaded with pyraclostrobin, as a targeted drug delivery system [45], phloem mobile derivative of fenpiclonil in combination with beneficial endophyte [44], and copper-based treatments, formulated with hydroxyapatite (HA) as co-adjuvant with innovative delivery properties [47,48] have shown promising results in controlling both esca and Botryosphaeria dieback pathogens. This last formulation has also shown an elicitation ability towards several genes related to plant defense [48]. However, increasing consumer demands for reduced chemical use and growing restrictions on the use of synthetic pesticides have increased interest in the use of natural active ingredients such as biocontrol agents (BCAs). Research on BCAs has greatly increased over the last years, being *Trichoderma* species the most studied as bio-pesticides [49]. Several *Trichoderma*-based products are currently used as pruning wound protectants against GTDs (for review see [1] and [28]) and several studies have been conducted using these products, showing encouraging results, against Botryosphaeriaceae species [28,40,50,51,52,53,54,55,56]. However, although several promising solutions currently exist, it seems impossible to manage Botryosphaeria dieback using only a single approach. An integrated pest management (IPM) strategy has been recommended including cultural practices, organic products, BCAs, responsible use of chemical fungicides, and control management that may combine both chemical and biological products [28,33].

To the best of our knowledge, no studies on strategies integrating pruning wound protection with chemical or biological products, and the application of site-targeted fungicides, with elicitor properties, have been conducted against Botryosphaeria dieback pathogens, on established vineyards. The main objectives of this work were thus to (i) test the efficacy of one *Trichoderma* based formulation, namely Esquive, single or in combination with LC2017, a new site-targeted copper-based treatment, formulated with hydroxyapatite loaded with copper (II) sulphate pentahydrate (CuSPHy + HA) which has a fungistatic and elicitor effect and, (ii) to compare their efficacy with a strategy of combining the application of commercially available chemical pruning wound protectant, Tessior^®^, a liquid polymer containing boscalid and pyraclostrobin alone and with LC2017, to prevent both infection and colonization by *Lasiodiplodia* spp. in field trials.

## 2. Results

### 2.1. In Vitro Assays

#### 2.1.1. Mycelial Growth Inhibition Assays

The inhibition of mycelial growth for all fungi under study ranged from 3.7–100% (Table 1). Significant differences were observed amongst several concentrations of fungicide used. Independently of the fungal species, the 12.5 mL/L concentration was able to completely inhibit mycelial growth. For all *Lasiodiplodia* spp. Isolates, the remaining concentrations were able to cause a similar growth inhibition rate. It is noteworthy that a significant decrease in mycelial inhibition percentage was verified from the highest concentration of fungicide (12.5 mL/L) to the second-highest concentration (2.5 mL/L) with inhibition percentage values decreasing from 100% to 14–15%. Higher inhibition percentages could be observed for *Trichoderma atroviride* strain I-1237, with a lower decrease between the two referred concentrations, from 100% to 44.6%. For the *Lasiodiplodia* spp. isolates, similar values were observed for the remaining concentrations. Moreover, for the three isolates, no significant differences were reported for the two lowest concentrations tested of 0.125 mL/L and 0.025 mL/L. *T. atroviride* strain I-1237 showed a slightly different behavior for the 1.25 mL/L concentration compared to the *Lasiodiplodia* spp. isolates, with a higher inhibition percentage, but showed similar behavior for the lowest concentration tested. In this case, *T. atroviride* strain I-1237 was the only fungi under study that showed significant differences for the inhibition percentage at the two lowest fungicide concentrations recording the second-lowest inhibition percentage for the 0.025 mL/L concentration. To verify if the effect observed on plates with 100% mycelial growth inhibition was fungistatic and not fungicidal, mycelium disks were transferred to fresh PDA and allowed to grow for 48 h. For all fungi, mycelial growth was observed with average values of 7.0 cm for isolate Bt105, 5.36 cm for isolate LA-SOL3, 4.54 cm for CBS124060, and finally 5.08 cm for *T. atroviride* strain I-1237. The renewed growth for all fungi is an apparent indicator that, during these in vitro assays, product LC2017 merely showed a fungistatic instead of a fungicidal effect.

#### 2.1.2. Dual Culture Antagonism Assays

**Figure 1:**
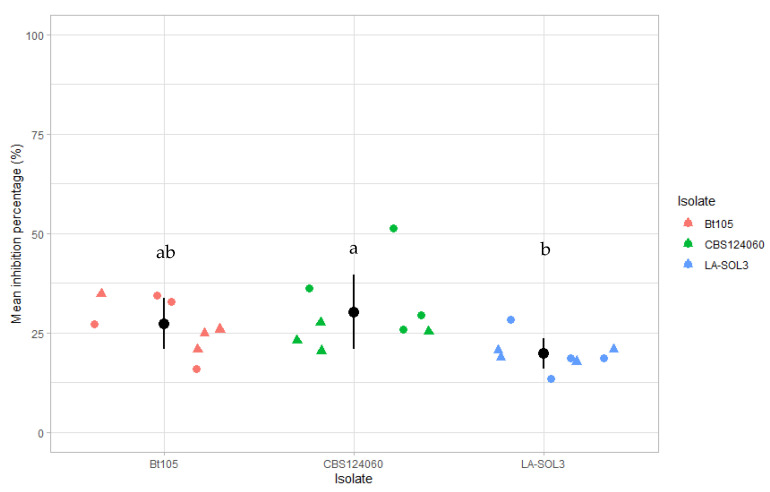
Mean inhibition percentage of growth radius for *Trichoderma atroviride* strain I-1237 against *Lasiodiplodia theobromae* isolates Bt105 and LA-SOL3 and *Lasiodiplodia mediterranea* isolate CBS124060. Dots and triangles represent data from both experiments, black dots represent the mean percentage of growth inhibition for the total replicates of both experiments and black bars represent the standard error of means. Columns with the same letter (a,b) are not significantly different according to Tukey’s test (*p* = 0.05).

**Figure 2:**
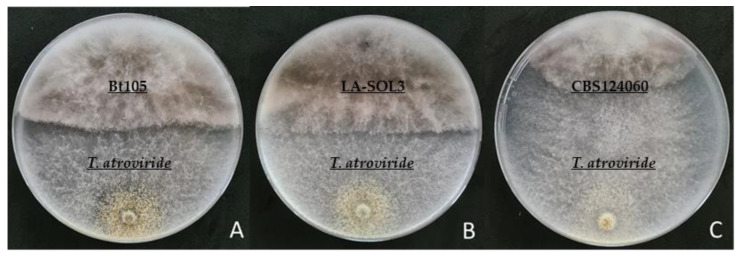
Dual culture antagonism assay of *T. atroviride* strain I-1237 and the three *Lasiodiplodia* spp. isolates under study. (**A**) isolate Bt105; (**B**) isolate LA-SOL3; (**C**) isolate CBS124060.

The levels of antagonism of *T. atroviride* strain I-1237 against the *Lasiodiplodia* spp. isolates evaluated, ranged from 14.0% to 51.2% (Figure 1). The lowest mean percentage of inhibition of radial mycelial growth was observed for isolate LA-SOL3 (19.8%) (Figure 2B) while the highest mean value of inhibition was recorded for isolate CBS124060 (30.2%) (Figure 2C). Significant statistical differences were observed between the mean inhibition percentage of radial growth of these two isolates. Isolate Bt105 showed values between the remaining two isolates with a mean inhibition percentage of radial growth of 27.3%, not significantly different from any of the referred isolates. Overall, the results show an apparent higher antagonism by *T. atroviride* strain I-1237 against the mycelial growth of the *L. mediterranea* isolate than for *L. theobromae* isolates (Figure 2).

### 2.2. Field Assays

The efficacy of the treatments was assessed based on the mean percentage of pathogen recovery (MPR) and, the mean percentage of disease control (MPDC) was calculated according to Sosnowski et al. [57,58] and Martínez-Diz, et al. [41]. Statistically significant differences were found amongst the treatments for both cultivars and years of the study (Table 2) when compared to the inoculated controls. For cultivar Cabernet Sauvignon, during the 2019 assay, treatments with Esquive + LC2017 reduced the MPR of isolate CBS124060 showing an MPDC of 70.8%, while Tessior + LC2017 was able to significantly reduce the MPR for both isolates Bt105 and CBS124060, with MPDCs of 81.5% and 75.0%, respectively. Tessior alone significantly reduced the MPR of isolate LA-SOL3 with an MPDC of 78.3%, and although an MPDC of 34.8% for isolate LA-SOL3 was recorded for the treatment with Esquive alone, no significant reduction was observed for any isolate. During 2020, a significant reduction in MPR of isolate Bt105 was verified for all the treatments, being the highest MPDC of 67.7% for the treatment with Tessior + LC2017. The only remaining significant reduction in MPR was on the pruning wound treatment with Tessior challenged with the isolate CBS124060 (MPDC 55%). Regarding cultivar Touriga Nacional, in 2019, no significant reduction in MPR was found for any treatment, when compared with the inoculated control, with the sole exception of Esquive inoculated with isolate CBS124060 (MPDC 53.3%). The 2020 assay showed a significant reduction in the MPR on treatment Esquive + LC2017 for isolates LA-SOL3 and CBS124060, with MPDCs of 48.4% and 58.6%, respectively. Isolate Bt105 provided a significant reduction in MPR with an MPDC of 52.5% on treatment with Tessior + LC2017, while treatment with Tessior was able to provide an MPDC of 45.2%. No significant reduction in MPR was verified for none of the remaining treatments when compared to the inoculated controls. Trichoderma spp. was only isolated from samples treated with Esquive. In 2019, recovery rates on Cabernet Sauvignon ranged from 24% to 16% for Esquive + LC2017 and Esquive treatments, respectively. For Touriga Nacional, the recovery rate for Esquive + LC2017 treatment was 20%, while a 30% recovery rate was recorded for Esquive applied alone. During 2020, Trichoderma spp. recovery rates for treatments with Esquive + LC2017 and Esquive alone were 38% and 28% for Cabernet Sauvignon and 25% and 22% for Touriga Nacional.

**Figure 3:**
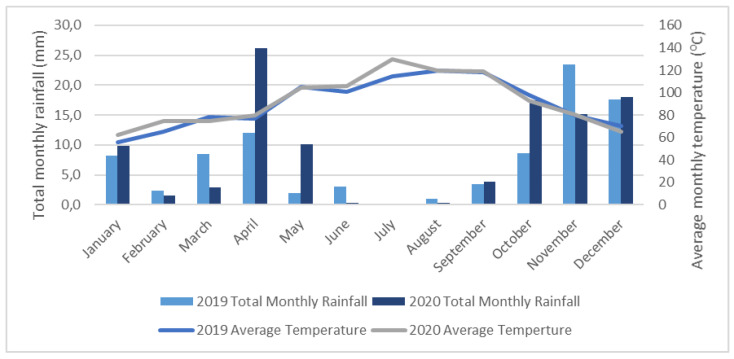
Monthly rainfall and average temperature for the two years of the trial.

Meteorological data recorded during the time of pruning treatments and pathogen inoculation for both years are presented in Figure 3. In 2019, the average daily temperature in the week of treatment and inoculation (11–15 February) was 10.05 °C with no rain events recorded. In 2020, treatments and pathogens inoculations were performed during the week from the 16–20 February, with an average temperature of 13.3 °C and only one rain event recorded on the 17th with 1.1 mm of precipitation. Regarding the total rainfall and average temperature for the month of February of both years, 2019 recorded an average temperature of 12.2 °C and a total precipitation of 12.7 mm spread throughout five rain events. In 2020, the average temperature was 14.0 °C, and a total precipitation of 8.4 mm with eight rain events.

**Figure 4:**
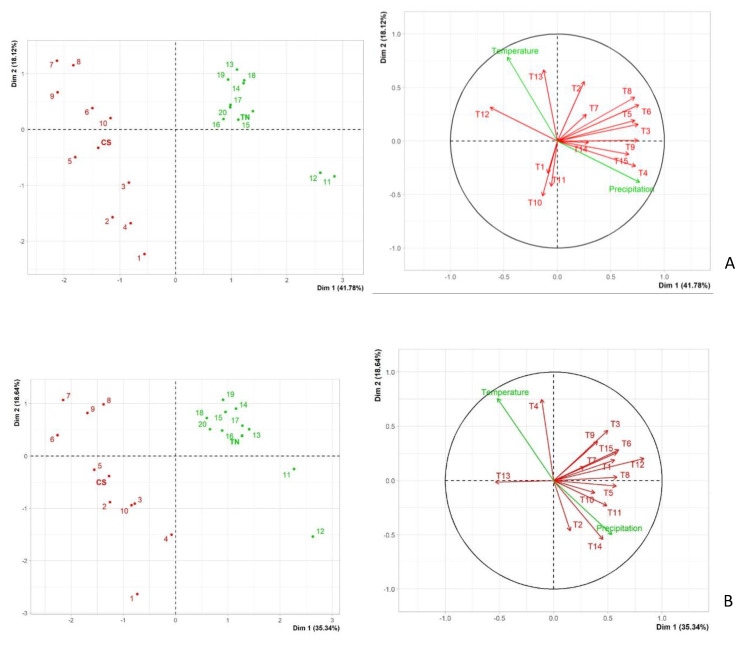
Principal component analysis (PCA) for the interactions of all treatments, 15 variables including all the product/isolate combinations (T represents the treatment numbers which are stated in Table 2), as well as inoculated controls on both variables and the meteorological variables (Figure 2). Red marks represent the observations for Cabernet Sauvignon (CS) and green marks represent the Touriga Nacional (TN) observations. (**A**)-PCA analysis for 2019; (**B**)-PCA analysis for 2020.

The principal component analysis (PCA) was performed to better visualize the relationship between all the variables (Figure 4A,B). For 2019 (Figure 4A), the two first principal components, accounted for 59.9% of the total variability of the data (PC1—41.78%; PC2—18.12%), while for 2020 (Figure 4B) the two first principal components represented 53.98% of the total variability of the data (PC1—35.34%; PC2—18.64%). For both years, we could also observe a clear distribution of the RP of the treatments according to the cultivar, which probably results from the influence of the different susceptibility that both cultivars may have towards the different pathogens studied. The relationship between the treatments and the meteorological variables could be observed in the vector plots of Figure 4A,B. For both years, meteorological variables showed similar behavior with precipitation being more related to component 1, while temperature showed a higher relation with component 2. In the vector plots, it was observed that the two meteorological variables were very weakly correlated, especially in 2020, where almost no correlation occurred between the variables. Since the vineyard was in an area with a Köppen climatic classification of Csa (hot-summer Mediterranean climate), it makes sense that these variables are negatively correlated since higher temperatures are equivalent to less precipitation. This is again evident for the lower correlation verified in 2020, as less rainfall was recorded during the summer months compared to 2019 (Figure 3). For 2019 (Figure 4A), most of the treatments showed a much higher correlation with precipitation than with the average temperature recorded throughout the duration of the assay. The only exception was for treatments 2, 12, and 13 which showed a higher correlation to the average temperature recorded. A negative correlation was verified between some treatments, such as T4 and T12 and, T2 and T10. T4 was a treatment with Tessior + LC2017 inoculated with isolate Bt105 while T12 was a treatment with Esquive inoculated with isolate CBS124060. This negative correlation may be due to not only these treatments having very different modes of action (chemical combination vs BCA), but also because the behavior of the two cultivars was completely opposite. T4 appeared to have higher efficiency than T12 on Cabernet Sauvignon, with MDPC values of 81.5% and 20% respectively, while the exact opposite was verified for Touriga Nacional, with 39.3% and 53.3% (Table 2). Treatments T2 and T10 also showed a negative correlation with each other, and although they are both treatments with Esquive (T2 is Esquive + LC2017 inoculated with isolate LA-SOL3 and T10 is Esquive applied alone inoculated with isolate Bt105) again the different efficacy of the treatments in both cultivars seems to have significance, since T2 seems to be more effective in Cabernet Sauvignon with higher MPDC values than T10, but the opposite was recorded for Touriga Nacional (Table 2). This trend is also verified for treatments T2 and T1, which also show a negative correlation. Both treatments are Esquive + LC2017 but inoculated with different isolates (T1-Bt105 and T2-LA-SOL3). MPDC values of T2 (26.1%) appear to show once again a higher efficacy in Cabernet Sauvignon than in Touriga Nacional (18.5%), while T1 appears to have a better impact in protecting Touriga Nacional (25%) than Cabernet Sauvignon (18%). This behavior of treatments with Esquive (alone or in combination) where T1 and T10 showed higher MPDC levels for Touriga Nacional comparatively to Cabernet Sauvignon was also verified for T11 and was confirmed by the high correlation showed by these three variables (Figure 4A). It is also noteworthy that all the treatments with chemical formulations, namely Tessior + LC2017 (T4, T5, T6) and Tessior applied alone (T7, T8, T9) showed high correlation amongst themselves, all being positively correlated with PC1. The only treatment with a BCA (Esquive +LC2017) showing a high correlation with the chemical-based ones was T3. The other *Trichoderma* treatments (Esquive + LC2017 and Esquive applied alone) showed mixed behavior, while the treatments T1, T10, and T11 were highly correlated with each other, but negatively correlated with T2 and almost uncorrelated with T12. For 2020 (Figure 4B), a similar behavior was observed for most of the treatments. Almost all the treatments showed a high correlation with precipitation, except for treatment 4, which revealed again a high correlation with precipitation during the previous year and for treatment 13 again. In this case, treatments 3 and 9 exhibited a similar correlation with both precipitation and temperature. Therefore, for both years of the assay, almost all isolates showed a higher correlation with precipitation rather than temperature, except for treatment T13 apparently more strongly related to temperature. This treatment also showed a negative correlation with T8, and very low to almost no correlation to the remaining treatments, which could be related to the fact that T13 corresponds to the inoculated control of isolate Bt105. During this year, treatments T2 and T4 showed a negative correlation. These are again treatments with different modes of action since T2 is a treatment of Esquive + LC 2017 inoculated with isolate LA-SOL3 and T4 is a treatment of Tessior + LC2017 inoculated with isolate Bt105. As was observed for the 2019 analysis, this negative correlation may be attributed to T2 being apparently slightly more effective in Touriga Nacional (MPDC = 58.6%) than in Cabernet Sauvignon (MPDC = 53.8%) while the opposite result is found for T4, with an MPDC of 67.7% in Cabernet Sauvignon opposed to 52.5% in Touriga Nacional. On this 2020 analysis, it is also noteworthy that all the treatments inoculated with isolate LA-SOL3 (T2, T5, T8, T11, T14) appear to have a high correlation amongst themselves, showing a low correlation with the remaining treatments. The exception was T5 which also showed a high correlation with the referred treatments, but nonetheless, this correlation shows that not only the variety and the type of product applied but also the isolate inoculated during this assay, may have some influence on the efficacy of the treatments tested.

## 3. Discussion

The present study reports the first field assessment on the efficacy of using management strategies, integrating biological and chemical pruning wound protectants with a copper-based site-targeted formulation, against Botryosphaeria dieback pathogens. Higher demand for more sustainable management practices and increasing restrictions on pesticide use has led to an expansion of IPM programs, involving organic products, BCAs, improved cultural practices, and responsible pesticide use [2,26]. To date, and to the best of our knowledge, no field assays have been performed using the two selected pruning wound protectants against *Lasiodiplodia* spp., in combination with copper-based protection with an elicitor effect. Our results show that the efficacy of the wound protectants, when applied alone or in combination with the copper-based product, varied between both years and according to the cultivar under study.

### 3.1. Efficacy of Pruning Wound Protection Products Applied Alone

For 2019, the highest MPDC values observed for Cabernet Sauvignon were found for the treatment with Tessior, while the lowest values were recorded for the application of Esquive alone. Nevertheless, the same pattern was not observed in 2020 when, in general, both treatments were able to reduce MPR to a similar extent. Pitt et al. [12] reported similar results in Australia, where liquid and paste formulations showed better efficacy against *Diplodia seriata* and *Diplodia mutila* than *Trichoderma*-based products. More recently, Martínez-Diz et al. [41] also described a higher efficacy of Tessior compared to Esquive, on pruning wounds infected with *D. seriata* and *Phaeomoniella chlamydospora*. A slightly different trend could be observed for cultivar Touriga Nacional in 2019 with the highest MPDC values for grapevines treated with Esquive alone. In 2020, this cultivar showed similar efficacy of the Esquive treatment comparing with the Tessior treatment, the only exception being the MDPC values found for isolate CBS124060 which were significantly lower than those reported for Tessior. In fact, all the 2020 treatments induced a slightly higher efficacy than observed in 2019, except for the Esquive treatment applied alone. Similarly, chemical fungicides, namely benomyl, were less effective than *Trichoderma* spp. treatments on wounds inoculated with *Diaporthe ampelina*, *D. seriata*, *E. lata*, *Neofusicoccum australe*, *Neofusicoccum parvum*, *L. theobromae* and *P. chlamydospora* [1,59]. This specific *T. atroviride* strain I-1237 has shown efficacy in reducing GTD incidence and severity in preliminary assays carried out in both Portuguese [55] and French [54] vineyards. Nevertheless, Martínez-Diz et al. [41] did not recently find significant differences between pathogen re-isolation from pruning wounds treated with *T. atroviride* strain I-1237 and inoculated non-treated controls.

The variable effectiveness of Trichoderma-based treatments has been previously reported by authors [34,41,51,60,61] and could be attributed to several reasons. The main advantage of using Trichoderma-based products as pruning wound protectants is the long-term protection conferred by the fungus growing in the wood. Therefore, the success of the protection provided by these products is dependent on the establishment of *Trichoderma* spp. within the wound. The influence of grapevine cultivar to wound colonization by *Trichoderma* spp. has also been highlighted by Mutawila et al. [52], probably related to the plant defense responses that differ between cultivars. These different defense responses to *T. atroviride* have been recently reported by Leal et al. [62], where the authors observed that *T. atroviride* may act as a priming stimulus for Tempranillo plantlets, while no stimulus could be verified for Chardonnay. This is in agreement with our PCA analysis which showed a negative correlation among treatments with *T. atroviride* (Esquive), likely due to a difference of efficacy amongst cultivars, which reinforces the importance of the cultivar in wound colonization by *Trichoderma* spp. It is known that not only cultivar but also meteorological conditions, such as temperature and precipitation, as well as the time of application influence the establishment and persistence of *Trichoderma* spp. [63]. Both cultivar and meteorological conditions factors could explain the differences of *T. atroviride* strain I-1237 colonization in our study. According to Esquive manufacturer, this specific *T. atroviride* strain I-1237 can grow at temperatures above 5 °C. In our study, the average temperatures recorded during pruning and treatments performed were 12.2 °C and 14 °C in 2019 and 2020, respectively. This may explain the apparent higher success on the colonization of the pruning wounds, especially verified for all the treatments with Esquive on Cabernet Sauvignon. Martínez-Diz et al. [41], while using the same commercial formulation of *T. atroviride* strain I-1237 (Esquive), found significantly lower colonization in Spanish vineyard trials. Nevertheless, this study was conducted on a different cultivar (Godello) and under different meteorological conditions. In this latter study, temperatures recorded during pruning and treatment application were significantly lower than those recorded during our study (6.5 °C and 9.8 °C in Spain to 12.2 °C and 14.0 °C in Portugal). The PCA analysis performed during this study also showed that most of the treatments where *T. atroviride* strain I-1237 was used were more correlated with precipitation than with temperature, being the only exceptions of treatments 12 and 13 in 2019. Nevertheless, in 2020, there were higher precipitation levels during the months of April and May, and on the PCA analysis performed for this year, all treatments with *T. atroviride* showed a high correlation with precipitation. This seems to reinforce the hypothesis that cultivar and meteorological conditions, especially rainfall, may apparently impact both the establishment and persistence of *T. atroviride* strain I-1237 in pruning wounds. As expected, for both treatments with Esquive, the highest percentages of colonization by *T. atroviride* strain I-1237 led to the highest mean percentage of disease control values. Further research is still needed to prove this hypothesis in GTDs which is characterized by extremely high complexity.

During our study, the application of Tessior (pyraclostrobin + boscalid + liquid polymer) was able to provide some reduction in infection by *Lasiodiplodia* spp. The highest mean percentages of disease control were obtained for Cabernet Sauvignon during 2019, with values as high as 78.3%, while during the next growing season, MPDC values only reached values as high as 55.5%. For Touriga Nacional, as referred to earlier, Tessior application was apparently not as effective as that of *T. atroviride*. The maximum level of MPDC was 45.2% for 2020, and MPDC values as low as 13.3% could be found during the previous year. Application of pyraclostrobin alone was effective in reducing infection by Botryosphaeria dieback fungi under field conditions [35,42], and a mixture of pyraclostrobin with metiram in the nursery when diluted in the soaking water prior to grafting [64]. Prior to this work, only preliminary studies and one in-depth study have been conducted by applying Tessior to pruning wounds, but all of them for controlling *Diplodia* spp. and *P. chlamydospora*. Preliminary field studies were conducted in Greece [65,66], Germany [64,67], and Spain [64] where Tessior was effective in reducing infection by the referred fungi. Recently, a more extensive study was conducted in Spanish vineyards by Martínez-Diz et al. [41] where Tessior showed a high MPDC for both *D. seriata* and *P. chlamydospora* compared to several other commercially available fungicides and BCAs. No previous studies have been conducted using Tessior as a pruning wound protectant against *Lasiodiplodia* spp., and although on the referred study, a botryosphaeriaceous fungi was used, *D. seriata* is considered a less aggressive species in comparison to *Lasiodiplodia* spp. [7]. In our study, we used two isolates of *L. theobromae* and one isolate of *L. mediterranea*. The establishment of the pathogens within grapevines depends on several factors, including not only meteorological variables and cultural practices, but also pathogen intrinsic properties such as aggressiveness [42]. Van Niekerk et al. [68] attributed the higher pathogen infection level on inoculated wounds to higher percentages of rainfall in the Stellenbosch area. This is in agreement with the PCA analysis of most of the treatments in our field study, which were strongly correlated with precipitation. The only treatment that for both years had a higher correlation with temperature was T13 corresponding to the inoculated control of isolate Bt105 (*L. theobromae*). It has been suggested that the expression of virulence factors in *L. theobromae* can be modulated by temperature [8,69]. This isolate was collected in Portugal, and it was previously considered to be highly virulent against both cultivars under study [70], suggesting that it may be better adapted to the local conditions than the other isolates under study. Moreover, our PCA analysis showed a high correlation between all the treatments in which plants were inoculated with isolate LA-SOL3 and between plants inoculated with isolate CBS124060 in 2020. Consequently, the difference in MPDC values found herein, comparatively to previous studies, may be due to the meteorological variables but also related to the pathogen used, and the difference in aggressiveness between them. Another factor that may be noteworthy is the difference in susceptibility between the two cultivars used. In fact, on average higher MPDC values were obtained for Cabernet Sauvignon during both growing seasons of the field assay, compared to the values found for Touriga Nacional. This tendency is verified, not only for the treatments with Tessior, but for most of the treatments. Previous studies conducted on the same cultivars using the same isolates, proved their high aggressiveness towards grapevine in both field and greenhouse assays [70], suggesting that the difference in efficacy in reducing infection by *Lasiodiplodia* spp. may also depend on cultivar susceptibility. Moreover, Sofia et al. [71] showed that from a set of four different cultivars, Touriga Nacional was one of the most susceptible to *P. chlamydospora*, suggesting that this particular cultivar may be highly susceptible to GTDs in general. Therefore, and given the differences found between both growing seasons and both cultivars, further research is recommended to evaluate each of the components of these products to understand how their efficacy may be affected by factors such as time of application, pathogen species aggressiveness and cultivar susceptibility, pruning wound size, and vineyard terroir.

### 3.2. Efficacy of a Strategy Combining the Application of Pruning Wound Protectants with LC2017

The great diversity of species currently associated with Botryosphaeria dieback, but also with all GTDs, combined with the intrinsic differences in hundreds of cultivars planted worldwide under different terroir conditions, makes the research for an effective management strategy to reduce the impact of GTDs an extremely difficult challenge. To this extent, in this work, we tried to integrate currently used products for pruning wound protection with the application of a site-targeted product based on a copper (II) compound (copper sulfate) and synthetic nanostructured particles of hydroxyapatite (HA) which have already shown interesting drug delivery properties in planta [46,47,48,72]. In our work, the application of this new site-targeted copper (II) formulation (LC2017), does not seem to greatly increase the efficacy of the pruning wound protection strategy. In some cases, the combination of the two strategies actually increased the mean percentage of disease control, but in others, the opposite was also verified. For example, in 2019, and for the inoculation of isolate CBS124060 on Cabernet Sauvignon, an MPDC of 70.8% was obtained for the treatment with Esquive + LC2017 while only 16.7% was recorded for the treatment with Esquive alone. During the next year (2020) the same value of 60% was found for both treatments. In 2019, on Cabernet Sauvignon, the application of Tessior + LC2017 caused also an MPDC of 81.5% for isolate Bt105, while only 37% was found for the treatment with Tessior alone. For the two other isolates under study, LA-SOL3 showed an increase in MPDC when Tessior was applied alone, while the isolate CBS124060 showed an opposite trend. Although the highest concentration of LC2017 used in the in vitro assay for the inhibition of mycelial growth showed fungistatic effect for all the isolates and *T. atroviride* strain I-1237, there is no apparent pattern on the influence of the LC2017 application as part of an IPM for any of the cultivars, or specific isolate. Moreover, the fungistatic effect verified for *T. atroviride* strain I-1237 did not seem to impact the colonization of pruning wounds by this BCA, since the highest values of re-isolation percentage could be found for the treatments combining Esquive and LC2017 except for Touriga Nacional during 2019. Moreover, no relevant correlation between the treatments using LC2017 was observed for both years by the PCA analysis, indicating a low influence of LC2017 application on the MPDC values found in this study.

The copper formulation used (LC2017) has already shown both fungistatic and fungicidal effects in vitro and *in planta* against *Phaeoacremonium minimum* [46]. Mondello et al. [48] have also reported that LC2017 showed the same in vitro fungistatic effect on *D. seriata* and *N. parvum* as it was verified for our *L. theobromae* isolates. The same authors described as well, the ability of this formulation to activate GTD-related plant defense reactions [48]. Di Marco et al. [73] also tested a copper formulation against *P. chlamydospora* and *P. minimum*, which revealed the ability to reduce conidial germination in in vitro assays but was not able to reduce *P. chlamydospora* colonization on young potted grapevines. Amposah et al. [36] tested a copper hydroxide formulation against Botryosphaeria dieback fungi, and were not able to obtain any significant control both in in vitro and in plant tests. More recently, Mondello et al. [48] performed greenhouse in planta assays that did not show significant differences in stem necrosis length of plants treated with LC2017 and inoculated with *D. seriata* and *N. parvum*. This agrees with our results since the same copper formulation used does not appear to provide a significant impact when combined with pruning wound protectants in controlling these specific Botryosphaeria dieback fungi. Therefore, further research is needed on testing all the components of this formulation, since HA applied alone has shown a non-fungitoxic and stimulant activity on *P. minimum* [48] and *Botrytis cinerea* [71]. However, the efficacy of copper (II) products has been previously related not only as a fungicide but also as an elicitor of some plant defense responses. Aziz et al. [74] observed this eliciting effect for CuSO4 sprayed on leaves, and Battiston et al. [48] have also shown that this formulation of CUSPHy was able to strongly induce several plant defense genes. Mondello et al. [48] also showed that LC2017 (HA + Cu (II)) had the same elicitation potential as BTH (*S*-methyl benzo (1,2,3) thiadizole-7-carbohthioate), a commonly marketed elicitor (BION^®^, Syngenta, France) by inducing genes related with chitinase and glucanase synthesis and also genes related to the biosynthesis pathways of several phenolic compounds.

Therefore, new methods to efficiently deliver fungicides to specific targeted areas of the plant are considered to have great potential to improve GTD control, and further research is needed to investigate not only the impact that these formulations may have on several GTD pathogens but also on the plant microbiome. Further long-term field trials should also be undertaken to investigate the effectiveness that a prolonged management control combining pruning wound protection and site-targeted fungicides might provide in controlling GTDs.

During this work, to ensure a proper establishment of infection with the fungi under study, approximately 2000 spores of each of the *L. theobromae* and *L. mediterranea* isolates were used to challenge each pruning wound. This represents a high inoculum pressure compared to the levels that pruning wounds are usually exposed to under natural field conditions. This suggests that due to the higher inoculum applied to the pruning wounds, the real efficacy of all the treatments tested against *Lasiodiplodia* spp. may have been underestimated [28].

In conclusion, this study demonstrated the field potential of pruning wound protection formulations to control *Lasiodiplodia* spp., applied alone or in combination with a novel site-targeted copper (II) formulation. The efficacy of the studied products differed between the two cultivars used and between the two growing seasons of the duration of the assay. Nevertheless, some measure of control was achieved for all treatments studied, despite the application of the copper-based product LC2017 not showing clear evidence of improving the control obtained by the pruning wound protection products applied alone. Still, this new site target copper-based product may prove to be a viable way of reducing the amount of copper applied for diseases management on vineyards, especially with further European Union restrictions on copper use in agriculture. Furthermore, the combination of effective pruning wound protection management by using a combination of a BCA with a more sustainable copper-based product has already proven to have interesting results not only as an elicitor by strongly inducing plant defense genes but also in the control of *P. minimum*, as well as *Plasmopora viticola*, may lead, in the long term, to healthier grapevines, which may reduce the expression of GTD symptoms in vineyards. However, this hypothesis still needs to be clarified by conducting studies using the same management strategy over a longer period of time, targeting not only other grapevine cultivars but also other Botryosphaeriaceae species. Thus, good pruning practices and wound protection combined with sustainable management strategies, such as biostimulants and host resistance inducers, can reduce the impact of Botryosphaeria dieback, not only by controlling pathogens already common in a certain wine region but also by making it difficult for the potential establishment of new species.

## 4. Materials and Methods

### 4.1. In Vitro Assays

#### 4.1.1. Mycelial Growth Inhibition Assay

Prior to field application of the LC2017 (CuSPHy + HA) product on the field, mycelial growth assays were conducted using three *Lasiodiplodia* spp. isolates, Bt105, LA-SOL3, and CBS124060 (Table 3). An assay was also performed using the *T. atroviride* strain I-1237 (Esquive^®^, product developed by Agrauxine S.A. and commercialized by Idai Nature S. L.), to test the compatibility between both products. To obtain the *T. atroviride* strain I-1237, a solution was made by directly suspending the Esquive^®^ product, on 100 mL of sterile distilled water. A 1.5 mL aliquot was transferred to a 90 mm Petri dish containing 20 mL of Potato-Dextro-Agar (PDA, Difco, Sparks, MD, USA), and spread onto the surface using a sterile plastic loop. Petri dishes were then incubated at 25 °C for 7 days in absolute darkness. For the mycelial growth assays, a stock solution of product LC2017 was made by suspending the product at the recommended field concentration (250 L/ha) to be applied immediately after pruning, in 1000 mL of sterile distilled water (SDW). Six different concentrations were made in SDW and added to 50 °C molten PDA, and 20 mL was poured into each 90 mm Petri dish, with six replicate plates allowed for each combination of LC2017 concentration and isolate (both *Lasiodiplodia* spp. and *T. atroviride* strain I-1237). The test range of LC2017 product concentration ranged from 0.025 to 12.5 mL L^−1^ and the six concentrations tested were evenly distributed across that range. Four hours after preparing the plates, 3 mm diameter discs were cut from the actively growing margin of one-week-old colonies all the isolates and placed on the center of each plate. Control plates contained only PDA. Plates were incubated at 25 °C for 48 h, in complete darkness, after which the two perpendicular diameters of the colonies were measured using a digital caliper. Mycelial growth inhibition (GI) was calculated according to Battiston et al. [46]: GI = [(DC − DO)/DC] × 100, where DC is the diameter of mycelial growth in the control plates and DO is the diameter of mycelial growth in treated plates. To establish if the effect of LC2017 on the tested fungi was only fungistatic, inhibited fungal disks were reinoculated onto fresh PDA plates and their growth revival was observed after 48 h.

#### 4.1.2. Dual Culture Antagonism Assay

Dual culture antagonism assays were also performed to evaluate the antagonistic capability of the *T. atroviride* strain I-1237 (Esquive) against the three *Lasiodiplodia* spp. isolates targeted for study (Bt105, LA-SOL3 and CBS124060), using dual culture assays [49,75]. *Trichoderma atroviride* strain I-1237 cultures used for this assay were obtained using the same method described for the mycelial growth inhibition assays. Mycelium plugs with 5 mm diameter of *T. atroviride* strain I-1237 and each *Lasiodiplodia* sp. isolate were cut from the actively growing margin of 3-day-old colonies, growing on PDA. Plugs were placed on opposite edges of 90 mm Petri dishes containing 15 mL of PDA. Plates were then incubated for 5 days in the dark at 22 °C. Each *Lasiodiplodia* sp. isolate was grown individually under the same conditions as control plates. Each combination *Trichoderma*/*Lasiodiplodia* spp. was replicated four times and the assay was performed twice. The percentage of mycelium growth inhibition was calculated using the formula, percent inhibition (PI) = [(B − A)/B] × 100 [42], where A is the radius of pathogen mycelium growth on the dual culture plates, and B is the radius of *Lasiodiplodia* spp. growth on the control plates.

### 4.2. Field Assays

#### 4.2.1. Experimental Field

The assay was conducted between 2019 and 2020, on an experimental vineyard located at Instituto Superior de Agronomia (ISA) (38°42′33.5″ N 9°11′15.8″ W) Lisbon, Portugal, planted in 1998. Two cultivars were used for this assay, Touriga Nacional (TN) and Cabernet Sauvignon (CS), both grafted onto 140 Ruggeri. Vines were trained as bilateral cordons. Traditional cultural practices in the vineyard were kept throughout the whole assay, and disease management followed an IPM. The products applied were selected with the care of not containing any substance that could casually interfere with the assay.

#### 4.2.2. Fungal Isolates Used and Inoculum Preparation

Two *L. theobromae* and one *L. mediterranea* isolates were used for this assay (Table 3*). Lasiodiplodia theobromae* isolates Bt105 and LA-SOL3, were collected in Portugal and Peru, respectively, and were stored at the culture collection of Instituto Superior de Agronomia. Both were isolated from grapevine wood showing symptoms of cankers and wood necrosis. The *L. mediterranea* isolate used is from the CBS culture collection from the Westerdijk Fungal Biodiversity Institute, in Utrecht, Netherlands, with the accession CBS 124060. Although *L. mediterranea* is currently not reported in Portugal, it has been previously reported in other European countries and so, one isolate of this species was also included in this study, not only for comparison but also to investigate the efficacy of the studied products towards this species. Isolates were maintained in PDA and transferred to Petri dishes with PDA to promote colony growth. Cultures were incubated at 25 °C in complete darkness for 8 days. After incubation, cultures were plated onto 6 mm Petri dishes containing 2% water agar with autoclaved pine needles (*Pinus pinea*) and incubated at 25 °C under fluorescent light for a 12 h photoperiod, to pycnidia sporulation [76,77,78]. On the day of the inoculation, conidia were harvested by collecting pycnidia formed on the pine needles to a 1.5 mL Eppendorf tube containing sterile distilled water (SDW), crushing them with the help of a pestle, followed by shaking the tube in a vortex for one minute. Spore suspensions obtained were filtered through cheesecloth and the concentration was adjusted to 1 × 10^5^ spores/mL with the use of a hemocytometer (Brand, Wertheim, Germany).

#### 4.2.3. Experimental Design and Treatment Plan

Products used in this assay can be found in Table 4. Esquive was applied according to label dosage and manufacturer’s instructions and Tessior is a formulation ready for application. LC2017 was applied with the timing and dosage recommended by the manufacturer (Table 4). A total of 15 treatments were set up on grapevines of both cultivars, Touriga Nacional and Cabernet Sauvignon in a completely randomized design, with 10 repetitions per treatment. One shoot was inoculated per grapevine making a total of 150 plants used from each cultivar. The combination of treatments and isolates inoculated can be found in Table 5.

#### 4.2.4. Product Application and Pathogen Inoculation

**Figure 5:**
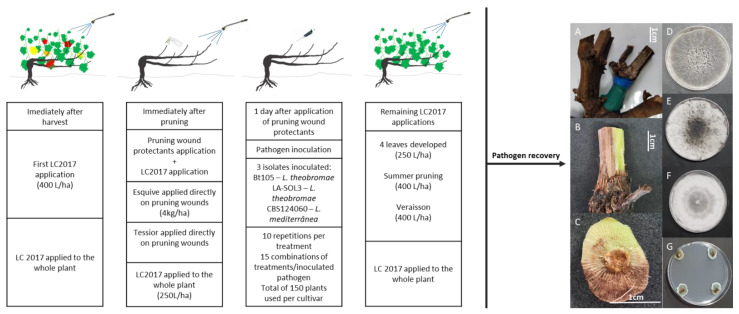
Diagram showing the several steps of the field assay. Information on the products used can be found in Table 4, and a description of the different treatments can be found in Table 5. (**A**) Marked sample collected from the field prior to analysis; (**B**) Sample with the bark removed showing sign of necrosis; (**C**) Sample collected about 1 cm below the pruning wound, to be divided into four pieces and plated onto PDA; (**D**) Isolate Bt105 (*Lasiodiplodia theobromae*) recovered from infected pruning wounds; (**E**) Isolate LA-SOL3 (*Lasiodiplodia theobromae*) recovered from infected pruning wounds; (**F**) Isolate CBS124060 (*Lasiodiplodia mediterranea*) recovered from infected pruning wounds; (**G**) Petri dish containing wood obtained from non-inoculated control plants showing no signs of pathogen growth.

For both seasons, immediately after harvest, one application of LC2017 was carried out as recommended by the manufacturer (Table 4; Figure 5). Harvest occurred during the month of September for both years of the assay. For both cultivars, one-year-old canes with a similar appearance, namely length, were selected for treatment followed by inoculation and were pruned at 2 cm above the third bud. After pruning, both products were prepared according to the label’s rate. Esquive was weighted and mixed with water in a 200 mL spray bottle with a concentration of 4 kg/ha (Figure 5). Tessior (ready to apply solution) and was applied using the equipment specially designed for this product application (Figure 5). Untreated controls were mock-treated with SDW, and the wound protectants were allowed to dry for a few hours. This was followed by application with LC2017, as indicated by the manufacturer (Table 4, Figure 5), using a backpack sprayer. One day after the treatment, inoculation with the selected was performed, by applying 20 µL of the spore suspension (≈2000 spores) on each wound using a micropipette (Figure 5). After inoculation, the pruning wounds were protected for one week using Parafilm M^®^ (Bemis, Sheboygan Falls, WI, USA) to prevent dehydration and promote spore germination. Pruning and artificial inoculation were performed on the 14 and 15 February 2019, and on the 20 and 21 February 2020, during the winter dormancy, taking into consideration that all the procedures were made during favorable meteorological conditions, namely cloudy and humid, but avoiding rain periods. The same precautions were taken for the remaining applications with LC2017, with the consideration of also avoiding days with strong winds to minimize spray drift.

#### 4.2.5. Pathogen Recovery and Identification

For both years, canes were recovered after harvest, during the month of October, and stored in a cold chamber (4 °C) until further processing. For pathogen re-isolation, the bark of each cane was removed, and a sample was collected from about 1 cm below the pruning wound (Figure 5). Four pieces of wood were collected from the border of necrotic internal tissue, surface disinfected with a 7% sodium hypochlorite solution, rinsed in SDW, and plated onto 9 mm Petri dishes containing PDA amended with chloramphenicol (PanReac, AppliChem, Darmstadt, Germany) at 250 mg/L. Plates were incubated at 25 °C, in the dark, and assessed for counting Botryosphaeriaceae and *Trichoderma* spp. colonies (Figure 5). A representative set of *Lasiodiplodia* spp. and *Trichoderma* spp. isolates was selected for identity confirmation. A DNeasy Plant Mini Kit from Qiagen^®^ (Venlo, The Netherlands) was used to extract genomic DNA from 8-day-old cultures grown in PDA and incubated at 25 °C, in the dark, following the manufacturer’s instructions. The identity of *Lasiodiplodia theobromae* and *L. mediterranea* was confirmed by sequencing part of the translation elongation factor 1α gene (tef1-α) by using the primers EF1-688F and EF1–1251R [79], while *T. atroviride* strain I-1237 was confirmed by sequencing the internal transcribed spacer region (ITS) using the universal primers ITS5 and ITS4 [80]. Amplified DNA was visualized on agarose gels stained with GreenSafe Premium (Nzytech, Lisbon, Portugal), and was visualized using a UV transilluminator to assess PCR amplification. PCR products were purified using an Illustra ExoProStar Enzymatic PCR and Sequencing Clean-up Kit (GE Life Sciences, Buckinghamshire, UK). PCR products were sequenced both ways at STABVIDA (Lisbon, Portugal) and compared with sequences from GenBank in BLAST searches.

#### 4.2.6. Meteorological Data

For both years of the experiment, daily temperature and rainfall were obtained from the Portuguese Institute for Sea and Atmosphere (IPMA—Instituto do Mar e da Atmosfera). These data were collected on the Lisbon reference meteorological station, which is located approximately 7 km from the vineyard tested.

#### 4.2.7. Statistical Analysis

All statistical analyses were performed using the R program (www.r-project.org (accessed on 30 September 2021)). The experimental data for both in vitro assays (mycelial growth inhibition and dual culture antagonism) were compared using an analysis of variance (ANOVA) followed by a Tukey’s test (*p* = 0.05). Prior to this analysis, Levene’s test was performed in order to verify the homogeneity of variance. Results of the dual culture antagonism assay were plotted using the R package ggplot2. The efficacy of the wound protectants was calculated as the mean percentage recovery (MPR) of the isolates under study. Normality and homogeneity of variance were tested using Levene’s test and when necessary, data were transformed into the arcsine of the square root of the proportion to verify the assumption of homogeneity of variance. For both years and cultivars, an ANOVA was used to compare the differences in the mean percentage of recovery (MPR). The means were compared using Tukey’s test at the 5% significance level (*p* = 0.05). The mean percentage of disease control was also calculated according to Sosnowski et al. [49,50] and Martínez-Diz et al. [41], using the formula MPDC = 100 × [1 − (MPR treatment/MPR inoculated control)]. To better visualize the results for all the treatments (15 variables, including all the product/isolate combinations, as well as inoculated controls) on both cultivars and their interaction with the meteorological variables (temperature and rainfall), a principal component analysis (PCA) was performed on the results of all the variables. For this analysis, data obtained from the treatments and meteorological variables were considered as two individual data sets or quantitative blocks, and cultivar was considered as a qualitative variable. This analysis was also performed using the R program with the Factoshiny v2.4 package [81].

## Figures and Tables

**Table 1:** Mycelial growth inhibition rate (%) of product LC2017 on all the *Botryosphaeriaceae* isolates under study (Bt105, LA-SOL3, CBS124060) and the *Trichoderma atroviride* strain I-1237 (Esquive).

LC 2017 Concentration	Mycelial Growth Inhibition (%) ^z^			
	Bt105	LA-SOL3	CBS124060	*T. atroviride* (I-1237)
A (12.5 mL/L)	100.0 ^a^	100.0 ^a^	100.0 ^a^	100.0 ^a^
B (2.5 mL/L)	14.5 ^b^	13.9 ^b^	15.0 ^b^	44.6 ^b^
C (1.25 mL/L)	10.4 ^c^	9.4 ^c^	10.6 ^c^	26.0 ^c^
D (0.25 mL/L)	8.0 ^cd^	6.2 ^d^	9.3 ^c^	9.9 ^d^
E (0.125 mL/L)	6.1 ^d^	5.1 ^d^	8.1 ^c^	9.2 ^d^
F (0.025 mL/L)	5.4 ^d^	3.7 ^d^	4.5 ^c^	3.9 ^e^

^z^ Values in the same column followed by the same letter (a, b, c, d, e) do not significantly differ according to Tukey’s test (*p* = 0.05).

**Table 2:** Efficacy of treatments used solely as pruning wound protectants and in a combination with the copper-based product, LC2017, to reduce *Lasiodiplodia* sp. as part of an integrated disease management.

Treatment	Product	Isolate	Cabernet Sauvignon				Touriga Nacional			
			2019		2020		2019		2020	
			MPR ^y^	MPDC ^z^	MPR	MPDC	MPR	MPDC	MPR	MPDC
1	Esquive + LC2017	Bt105	55.0 ^abc^	18.5	40.0 ^bc^	48.4	52.5 ^abc^	25.0	57.5 ^abc^	9.0
2	Esquive + LC2017	LA-SOL3	42.5 ^abcde^	26.1	30.0 ^bc^	53.8	57.5 ^abc^	8.0	30.0 ^c^	58.6
3	Esquive + LC2017	CBS124060	17.5 ^de^	70.8	20.0 ^c^	60.0	60.0 ^abc^	20.0	40.0 ^bc^	48.4
4	Tessior + LC2017	Bt105	12.5 ^e^	81.5	25.0 ^c^	67.7	42.5 ^bc^	39.3	30.0 ^c^	52.5
5	Tessior + LC2017	LA-SOL3	22.5 ^bcde^	60.9	45.0 ^bc^	30.8	60.0 ^abc^	4.0	60.0 ^abc^	17.2
6	Tessior + LC2017	CBS124060	15.0 ^de^	75.0	35.0 ^bc^	30.0	57.5 ^abc^	23.3	62.5 ^ab^	19.4
7	Tessior	Bt105	42.5 ^abcde^	37.0	37.5 ^bc^	51.6	52.5 ^abc^	25.0	42.5 ^bc^	32.8
8	Tessior	LA-SOL3	12.5 ^e^	78.3	40.0 ^bc^	38.5	50.0 ^abc^	20.0	55.0 ^abc^	24.1
9	Tessior	CBS124060	20.0 ^cde^	66.7	22.5 ^c^	55.0	65.0 ^abc^	13.3	42.5 ^bc^	45.2
10	Esquive	Bt105	62.5 ^ab^	7.4	35.0 ^bc^	54.8	50.0 ^abc^	28.6	40.0 ^bc^	36.7
11	Esquive	LA-SOL3	37.5 ^abcde^	34.8	40.0 ^bc^	38.5	32.5 ^c^	48.0	52.5 ^abc^	27.6
12	Esquive	CBS124060	50.0 ^abcd^	16.7	20.0 ^c^	60.0	35.0 ^c^	53.3	65.0 ^ab^	15.0
13	Inoculated Control	Bt105	67.5 ^a^	-	75.0 ^a^	-	70.0 ^ab^	-	65.0 ^ab^	-
14	Inoculated Control	LA-SOL3	57.5 ^abc^	-	65.0 ^ab^	-	62.5 ^abc^	-	72.5 ^a^	-
15	Inoculated Control	CBS124060	60.0 ^abc^	-	50.0 ^abc^	-	75.0 ^a^	-	77.5 ^a^	-

^y^ Efficacy of all the treatments based on the mean percentage of recovery (MPR) of all the isolates used in this study, from the inoculated pruning wounds. Values in the same column with the same letter (a, b, c, d, e) do not significantly differ according to Tukey’s test (*p* = 0.05). ^z^ Mean percentage of disease control (MPDC) of all the treatments calculated according to the formula MPDC = 100 × [1−(MPR treatment/MPR inoculated control)].

**Table 3:** *Lasiodiplodia* spp. isolates used for pruning wound inoculation.

Species	Isolates	Geographic Origin
*L. theobromae*	Bt105	Alentejo, Portugal
	LA-SOL3	Sol Sol, Piura, Peru
*L. mediterranea*	CBS 124060	Sicily, Italy

**Table 4:** Treatments tested for control of *Lasiodiplodia theobromae* and *Lasiodiplodia mediterranea* under field conditions.

Product Name	Manufacturer	Application Time	Application Rate	Active Ingredient
Esquive^®^	Idai Nature	After pruning	4 kg/ha	*Trichoderma atroviride* strain I-1237 (1 × 10^8^ CFU g^−1^)
Tessior^®^	BASF Agricultural Solutions Portugal	After pruning	n/a	Pyraclostrobin 0.48% + boscalid 0.95%
LC2017	Natural development Group^®^	Immediately after harvest	400 L/ha	Hydroxyapatite (HA) loaded with cooper (II) sulphate pentahydrate (CuSPHy + HA)
		After pruning (Winter)	250 L/ha	
		Four leaves developed	250 L/ha	
		Summer pruning	400 L/ha	
		Veraisson	400 L/ha	

**Table 5:** Treatment plan designed to assess the efficacy of three products against pruning wound infection by *Lasiodiplodia theobromae* and *Lasiodiplodia mediterranea*. Combinations of products used and inoculations spore solution volume.

Treatment	Product	Inoculation	Spore Solution Volume (µL)
1	Esquive + LC2017	*L. theobromae* (Bt105)	20
2	Esquive + LC2017	*L. theobromae* (LA-SOL3)	20
3	Esquive + LC2017	*L. mediterranea* (CBS124060)	20
4	Tessior + LC2017	*L. theobromae* (Bt105)	20
5	Tessior + LC2017	*L. theobromae* (LA-SOL3)	20
6	Tessior + LC2017	*L. mediterranea* (CBS124060)	20
7	Tessior	*L. theobromae* (Bt105)	20
8	Tessior	*L. theobromae* (LA-SOL3)	20
9	Tessior	*L. mediterranea* (CBS124060)	20
10	Esquive	*L. theobromae* (Bt105)	20
11	Esquive	*L. theobromae* (LA-SOL3)	20
12	Esquive	*L. mediterranea* (CBS124060)	20
13	Inoculated non treated Control	*L. theobromae* (Bt105)	20
14	Inoculated non treated Control	*L. theobromae* (LA-SOL3)	20
15	Inoculated non treated Control	*L. mediterranea* (CBS124060)	20
